# Long-Term Evaluation of the Use of the Transdermal Contraceptive Patch in Adolescents

**DOI:** 10.1100/tsw.2004.107

**Published:** 2004-07-08

**Authors:** Stephanie Logsdon, Jessica Richards, Hatim A. Omar

**Affiliations:** Department of Pediatrics, University of Kentucky, Lexington, USA

**Keywords:** contraception, adolescents, hormonal contraception, transdermal contraception, adolescent sexuality, adolescent pregnancy, United States

## Abstract

The transdermal contraceptive patch, Ortho Evra™, was approved in December 2001 and released on the market in June 2002. In this study, we reviewed clinical data of young women who started the patch between June 2002 and December 2003 in the adolescent medicine clinic at a university-based outpatient center. A total of 62 patients started the patch in that period and two of them were lost to follow-up. Mean age of patients was 17.9 years and mean length of use was 10 cycles. Only 10 patients (16.7%) discontinued use. Reasons for discontinuation were moderate to severe skin irritation (3 patients, 5%), complete detachment (3 patients, 5%), and economic reasons (4 patients, 6.7%). Compliance was excellent overall and the side-effects profile was good. No pregnancies occurred during this period. These results confirmed that the transdermal contraceptive patch is easy to use and an effective method of birth control that may be better tolerated by young women. It also seemed to improve contraceptive compliance in this population.

## INTRODUCTION

Over 12 million women in the U.S. and over 100 million women in the world use hormonal contraception[1]. Despite that, more than one-half of women with unintended pregnancies have reportedly used some form of birth control during the month of conception[2]. Using oral contraceptive pills in adolescents produces a failure rate of 5–18%[3,4,5]. The recently developed contraceptive transdermal patch, Ortho Evra™, by R.W. Johnson Pharmaceutical Research Institute, delivers norelgestromin and ethinyl estradiol to the circulatory system without going through the digestive tract and without the customary peaks and valleys associated with oral contraceptives[6]. Previous studies have shown that the serum concentrations of the hormones in the patch are within the reference ranges throughout the duration of adhesion, regardless of site application[7].

Ortho Evra$^{TM}$ is a patch comprised of three layers. The first layer is an outer protective layer made of polyester, the second is a medicated adhesive middle layer, and the third is a clear, polyester liner that is removed before patch application. The use of the patch is similar to oral contraceptives. One patch is applied to one of four sites on the body: the buttocks, arm, torso, or abdomen. This patch is left untouched for 7 days. At the end of 7 days, the patch is removed and a new one attached to a similar site on the skin. At the end of 3 weeks, with the removal of an old patch and application of a new patch each week, the user does not wear a patch for 1 week. This is the week where menstruation should occur. Each patch should be worn for no longer than 7 days. No creams, oils, or cosmetics should be placed near or on the application site[8].

The patch is user directed and compliance easier than oral contraceptives. The patch is also more readily reversible than longer-acting contraceptives and has a more convenient dosing schedule. Previous studies have found that compliance with the patch is significantly higher than with oral contraceptives[6]. The overall and method-failure rates were also found to be similar to oral contraceptives[1,8,9]. However, contraceptive failures may increase for women weighing more than 90 kg (198 lbs)[9]. Perfect compliance with the patch has been found to be more easily attainable by patch users. Also, compliance with the patch is uniform across all age groups, while compliance with oral contraceptives varies between age groups. Oral contraceptive studies have shown that younger users have a higher failure rate than older users[10]. Site selection and climate conditions did not appear to alter the phamacokinetics of the patch[7,11,12].

Side effects from the patch have been found to be similar to those associated with oral contraceptives. Breast discomfort, breakthrough bleeding, and spotting are slightly more common in the first two cycles with patch use. However, after the second cycle, the rates of these side effects are similar to oral contraceptives. Dysmenorrhea is also more common in patch users. Previous studies have found that breast discomfort, application site reactions, upper respiratory infections, dysmenorrhea, and headache can occur in over 10% of patch users. Most of the side effects were described as mild to moderate in severity[1].

In this study, we reviewed the use of Ortho Evra$^{TM}$ by young women over an extended period of time to assess compliance, side effects, efficacy, and discontinuation rates.

## METHODS

Medical records of young women, who started using the transdermal contraceptive patch between June 2002 and December 2003 in the Adolescent Medicine Clinic at the University-Based Health Center, were reviewed. Data were collected for compliance, side effects, discontinuation, and efficacy. Demographic data were also collected. Patients in this clinic were followed every 3 months to encourage compliance and to address concerns whenever they were on any form of contraception. Patients are provided with extensive counseling prior to starting any method of contraception. This counseling includes education on how to use that method, what to anticipate in terms of possible side effects, what to do if there is a problem, and counseling on sexually transmitted infections.

## RESULTS

A total of 62 patients chose to start using the transdermal contraceptive patch, Ortho-Evra$^{TM}$, during that period. The mean age of the patients was 17.9 years with a range of 13–23 (Table 1). Other demographic characteristics are detailed in Table 1. Two of these patients were lost at follow-up and not included in the study. Average length of use of Ortho-Evra$^{TM}$ was 10 cycles for a total of 600 cycles with a range of 4–18 months. Patients were revaluated every 3 months. At total of 10 patients discontinued use. Three patients

**TABLE 1**
**Demographic and Clinical Profiles of Patients**

| | |
|---|---|
| Age (Mean ± SD) | 17.5 ± 2 Years, Range 13–23 |
| Race | 56 W, 44 B |
| Menarche | 11.3 ± 1.6 Years of age |
| Initial BMI | 22.3 ± 4.1kg/m$^2$ |
| Previous pregnancy | 36% Yes, 64% No |
| Current sexual activity | 88.3% Yes, 11.7% No |
| Ever sexually active | 96.7% Yes, 3.3% No |
| Used previous hormonal method | 78.3% Yes, 21.7% No |
| Insurance | 60% MC, 29% Private, 11% None |

(5%) discontinued because of full detachment, another three patients (5%) discontinued because of moderate to severe skin irritation or allergic reaction, and four patients (6.7%) discontinued because of economic reasons (no insurance). Thirty patients (50%) are currently in their second year of use. Review of side effects showed skin irritation to be the most common complaint (13 patients, 21.7%) followed by partial or complete detachment (5 patients, 8.3%), while other side effects were less common (Table 2). As of the date of the data collection, no pregnancies were reported among the participants.

**TABLE 2**
**Reported Side Effects**

| | |
|---|---|
| Breakthrough bleeding | 2 (3.3%) |
| Amenorrhea | 2 (3.3%) |
| Recurrent headaches | 0 (0%) |
| Breast tenderness | 2 (3.3%) |
| Nausea | 6 (10%) |
| Weight gain | 4 (6.7%) |
| Detachment (partial or complete) | 5 (8.3%) |
| Skin irritation | 13 (21.7%) |

## DISCUSSION

Several studies have evaluated compliance, efficacy, and side-effects profile of the patch mostly in adult women[1,6,9,10,11,12]. To the best of our knowledge up to this point, there were only two studies in adolescents[13,14]. The two studies in adolescents showed results of brief periods of use (3 and 8 months, respectively). In this study, we evaluated patients who have used the patch for up to 18 months. One of the major concerns in adolescents is compliance with other birth control methods, with significant failure rates on oral contraceptive pills most likely due to noncompliance[3,4,5]. The ease of use of the contraceptive patch appeared to promote better compliance[8]. The side effects from other studies in adolescents also showed a good side-effect profile and good tolerability of the patients to these side effects.

Our results are similar to these found in adult women in terms of compliance, efficacy, and tolerability[1,6,9,10,11,12]. However, in our patient population, the discontinuation rate and the frequency of side effects differed slightly from those reported by others in the same age group[13,14]. Efficacy was excellent with no pregnancies reported and method-related discontinuation was only 10%, mostly due to detachment or skin irritation. Another group of patients (6.7%) discontinued use because of economic reasons. Side effects reported by our patients also differed somewhat from the other two studies[13,14]. Our patients reported a very low level of breakthrough bleeding, headaches, breast tenderness, and nausea compared to the above-mentioned studies[13,14]. The excellent compliance, low discontinuation rate, and lower reports of side effects may be due to the extensive counseling, thorough education, and frequent follow-up of these patients in our program.

Limitations in our study were the retrospective nature of the study and the relatively small number of participants. However, the number of participants is larger than the other studies reported in adolescence and the length of time of follow-up was much longer.

## CONCLUSION

Based on the results of this study and other studies reported in literature, the transdermal contraceptive patch was found to be an effective, well-tolerated, and easy-to-use method, which may help to improve long-term compliance in young women and reduction of teen pregnancy.

## BIOSKETCHES

**Stephanie Logsdon**, is an undergraduate student at the University of Kentucky, majoring in biology. She has worked during her summer research project with the Section of Adolescent Medicine, Department of Pediatrics, University of Kentucky, Lexington.

**Jessica Richards, RN**, Section of Adolescent Medicine, Department of Pediatrics, University of Kentucky, Lexington. She is very active in research concerning adolescents and teen mothers.

**Hatim A Omar, MD**, Professor of Pediatrics, Obstetrics and Gynecology and Director of the Section of Adolescent Medicine, Department of Pediatrics, University of Kentucky, Lexington. Dr. Omar has completed residency training in Obstetrics and Gynecology as well as Pediatrics. He has also completed fellowships in Vascular Physiology and Adolescent Medicine. He is the recipient of the Commonwealth of Kentucky Governor's Award for Community Service and Volunteerism and is well known internationally as an authority in Adolescent Medicine and Pediatric and Adolescent Gynecology. E-mail: haomar2@uky.edu
